# Exploring Common and Novel Actualized Affordances of Fitbit: Mixed Methods Study

**DOI:** 10.2196/85412

**Published:** 2026-02-18

**Authors:** Moayad Alshawmar, Bengisu Tulu, E Vance Wilson, Adrienne Hall-Phillips, Ibrahim Aljadani, Emmanuel Agu

**Affiliations:** 1 Imam Mohammad ibn Saud Islamic University (IMSIU) Riyadh Saudi Arabia; 2 Worcester Polytechnic Institute Worcester, MA United States

**Keywords:** fitness apps, exercisers, affordance, affordance actualization, exercise behaviors

## Abstract

**Background:**

Although fitness apps could promote healthier lifestyles, evidence on the effectiveness of app-based interventions remains inconsistent. Previous studies have used affordance theory to identify the factors that generate exercise-related value for users. However, many fitness app affordance studies have examined multiple fitness apps collectively, assuming similar design intentions across platforms. Moreover, most have relied on predefined affordances rather than investigating emergent or novel ones that may reveal unique user–fitness app interactions.

**Objective:**

This study aimed to identify the common affordances actualized by Fitbit users and uncover novel affordances that emerge from their interactions with this specific app, thereby extending the understanding of how affordances contribute to user engagement and health outcomes.

**Methods:**

We used a 2-stage mixed methods design. First, a cross-sectional web-based survey was conducted with 442 US-based Fitbit users engaging in regular exercise. The participants selected affordances from a list identified in prior literature and could report additional affordances in open-text responses. To corroborate and extend the survey findings, 15,000 user reviews were collected from the Google Play Store, of which 2674 (17.8%) comments were automatically categorized into affordance themes and 1182 (7.9%) were manually validated as relevant. Reviews were thematically classified into affordance categories via a generative pretrained transformer–based approach guided by survey-identified affordances.

**Results:**

The survey revealed that the most frequently actualized affordances were updating (351 participants and 749 review mentions; total=1100) and reminding (319 participants and 143 mentions; total=462), underscoring Fitbit’s role in tracking progress and sustaining routines. Competing (99 participants and 88 mentions; total=187) and rewards (133 participants and 32 mentions; total=165) highlighted gamification, whereas comparing (151 participants and 8 mentions; total=159) and guidance (118 participants and 25 mentions; total=143) reflected benchmarking and instructional support. Other affordances such as searching (135 participants and 2 mentions; total=137), encouraging (75 participants and 19 mentions; total=94), and watching others (68 participants and 3 mentions; total=71) were less common, whereas recognizing (58 participants and 0 mentions; total=58) and self-presentation (47 participants and 1 mention; total=50) were the least common. The novel affordances included encouraging others (14 participants and 1 mention; total=15), accountability (3 participants and 9 mentions; total=12), and self-comparison (3 participants and 5 mentions; total=8).

**Conclusions:**

Most Fitbit users actualized updating and reminding affordances, whereas a limited number of users actualized the other affordances. Moreover, few Fitbit users actualized novel affordances that reflect self-regulation, an extension of social connection, and personal meaning. This study emphasizes that Fitbit should focus on core tracking and reminding for most users while providing optional features that foster guidance, community, accountability, and personal relevance. Designing features that facilitate the actualization of common and novel affordances may improve app effectiveness and, ultimately, the health benefits of fitness technologies.

## Introduction

Mobile fitness apps have emerged as widely adopted digital tools intended to support individuals in pursuing healthier lifestyles [1]. Through their interactive and data-driven nature, fitness apps encourage consistent exercise, potentially enhancing both physical fitness and cognitive health while contributing to the prevention of chronic illnesses [2-4].

Although these apps hold potential for improving exercise behaviors, evidence on the effectiveness of app-based interventions remains inconsistent [5]. Several studies report minimal to no observable improvements in exercise following app-based interventions [6,7], whereas others demonstrate meaningful gains in exercise engagement and outcomes among adults [8,9]. These mixed findings indicate that the effectiveness of fitness app interventions in promoting exercise is not yet definitively established, highlighting the need to better understand the pathways through which these apps contribute to exercise improvement.

To clarify these inconsistencies, recent research has shifted from solely examining intervention health-related outcomes to investigating the factors that generate exercise-related value for users [4,10-12]. Some of these studies interpret fitness app use through the lens of technology affordance actualization, emphasizing what the app enables users to do rather than the app feature list. For example, while some fitness apps offer group-based exercise features, certain users may actualize them as sources of exercise guidance, whereas others may use them primarily for comparing themselves to other participants. This view recognizes that users may use apps in ways that either follow or depart from the purposes intended by the designers [13,14]. Thus, studying the apps’ affordances can help explain the core benefits of using apps from the users’ perspective despite their design differences because affordances are driven by users’ goals and how users interact with the app to achieve these goals.

Prior fitness app research has identified several affordances that users actualize while interacting with app features, such as self-monitoring, exercise guidance, competition, and social comparison [10,11,15-17]. However, many of these studies have analyzed multiple fitness apps collectively, with the assumption of shared design purposes across platforms. While such breadth may provide useful generalizations for some studies’ purposes, it risks overlooking meaningful differences rooted in users’ interaction with specific fitness apps that differ in design, even among apps with similar functions. For example, although Fitbit (Google LLC) and MyFitnessPal (MyFitnessPal, Inc) offer similar features (eg, activity tracking), we cannot assume that they are identical or that their users behave in the same way. What each app measures, how it tracks those measurements, and how the data are presented to users can differ substantially.

Furthermore, most prior fitness app research has focused on applying the literature-uncovered affordances rather than exploring new ones [15,16,18-20]. This approach may limit opportunities to discover new affordances, particularly given that affordance theory suggests that users actualize possibilities based on their diverse goals, needs, and intentions [13,14,21]. While applying literature-identified affordances can deepen our understanding of how these affordances contribute to exercise improvement, exploring new affordances can broaden research possibilities and provide developers with clearer insights into what their apps are used for.

This study aims to address these limitations by investigating both common (fitness app literature–predefined) affordances focusing their actualization on one fitness app (Fitbit) and novel (not yet uncovered) affordances actualized by users of the app. Specifically, we aimed to answer the following research questions: (1) which of the fitness app literature affordances are Fitbit app users actualizing? (2) What are the novel affordances actualized through users’ interactions with Fitbit?

This study used a 2-stage methodology to uncover the actualized affordances of the Fitbit app. First, a survey was conducted to identify both common and novel affordances experienced by users. On the basis of the survey findings, user reviews from the Google Play Store were analyzed and thematically classified via a generative pretrained transformer (GPT)–based approach guided by the affordances identified in the survey.

## Methods

### Study Design

This study used a cross-sectional web-based survey, and its design and findings were reported in accordance with the CHERRIES (Checklist for Reporting Results of Internet E-Surveys) guidelines [22]. The survey investigated the common and novel affordances actualized through user interaction with a widely used fitness app, Fitbit. To complement the survey, user reviews from the Google Play Store were analyzed. The affordances identified in the survey, both common and novel, were used as thematic categories to classify review comments through a GPT-based approach. By triangulating survey data with a large corpus of user reviews, we sought to establish robust evidence of the affordances actualized by Fitbit users.

### Measures

#### Survey

To uncover the common affordances actualized by Fitbit users, participants were provided with a list of established fitness app affordances, as shown in Table 1, and asked to select all that applied to their use of the app. Each affordance was accompanied by a short definition adapted from prior studies [11,17]. The participants could select one or multiple affordances that Fitbit enabled during their use.

**Table 1:** Survey items used to uncover the actualization of common fitness app affordances in the context of Fitbit.

Affordance		Definition
**What did you use Fitbit for? Choose all that apply.**		
	Comparing	To compare my exercise performance with others
	Guidance	To get guidance how to perform my exercise
	Self-presentation	To present myself as a physically active person
	Rewards	To receive rewards for my exercise
	Recognizing	To get recognition from others for my exercise
	Encouraging	To have other people encourage my exercise
	Competing	To compete with others
	Watching others	To keep an eye on others’ ways of doing exercise
	Reminding	To remind me to do an exercise activity
	Updating	To update me with the status of my exercise progress
	Searching	To search for exercise information

To capture novel affordances, the survey included an “other” option that allowed participants to write in additional action possibilities not included in the predefined list. These written responses were analyzed to determine whether they represented true affordances, that is, goal-directed action possibilities emerging from user-technology interaction rather than simple descriptions of Fitbit features. In doing so, we applied the 3 affordance identification principles described by Alshawmar et al [23], which are grounded in established affordance theory [13,21,24]. First, affordances must reflect a user-perceived action possibility. If a response did not contain a user goal or intended action, it was treated as a feature rather than an affordance. Second, affordances must be described in terms of *why* the user engages with the technology, not *what* the technology provides. For example, the ability to share achievements is a feature; “receiving encouragement from others” is an affordance because it reflects a purpose or goal behind using that feature. Third, multiple features may enable the same affordance. Therefore, responses were grouped at the level of user goal rather than specific app components.

All written responses were independently reviewed by 2 coders trained in affordance theory. Coding disagreements were discussed and resolved through consensus. Interrater agreement before reconciliation was 0.92, indicating high reliability. Responses that reflected affordances already included in the predefined list were classified as common and removed from this step. The remaining responses were examined for thematic similarity, which resulted in the identification of 3 novel affordances. Each novel affordance was then defined based on prior literature to ensure conceptual clarity. For example, written responses such as “to hold myself accountable” were classified as the novel affordance accountability, consistent with the definition of accountability by Dhiman et al [25] as the need to justify one’s actions to oneself.

In addition, demographic information and use data (eg, age, gender, duration and frequency of use, and premium subscription) were collected to provide context for interpreting users’ affordance actualization patterns.

#### User Review Data

To support the survey findings of common and novel affordances, we analyzed Fitbit app users’ review comments. User reviews were collected from the Google Play Store via an automated web scraping tool [26]. Approximately 380,000 reviews were initially gathered spanning multiple app versions and time ranges. After preprocessing and filtering, we removed duplicates, non–English-language content, very short reviews with fewer than 6 words, and reviews retained from the past 3 years that contained the word “to” as a simple heuristic for goal-directed app use (eg, “to track progress” and “to stay updated”). Standard text-cleaning steps such as lower casing and punctuation removal were applied to prepare the data for analysis. This data preprocessing resulted in a final sample of 15,000 reviews.

For thematic classification, we developed a model based on 14 predefined affordance-based themes identified from prior literature and the survey findings. Each review was analyzed using GPT-4.1 mini (OpenAI) via an instruction-based, zero-shot, label-constrained prompt designed for thematic classification (refer to Multimedia Appendix 1 for more details about the prompt).

The prompt introduced the model to the predefined affordance categories, each clearly defined and exemplified (eg, “Comparing; comparing performance with others” and “Guidance; receiving instructions on how to exercise”). The GPT was instructed to select exactly 1 theme per review when applicable or to generate a single-word new (nonaffordance) theme if the review did not fit any predefined category. The large language model’s temperature was set to 0.3 to minimize variability and ensure stable classifications across reviews [27,28].

This resulted in a dataset consisting of 12,378 reviews categorized as “nonaffordance” and 2622 reviews assigned to the predefined affordance themes.

This design encouraged the model to apply conceptual reasoning rather than keyword matching, aligning the classification with the theoretical framework of affordance actualization (refer to the example classifications in Multimedia Appendix 2).

To ensure classification accuracy, human validation of the GPT-based thematic assignments was conducted. After the GPT classified the full dataset, a stratified random sample of 300 reviews was drawn. One author manually coded the validation sample, applying the same thematic definitions provided to the language model. To assess agreement, the author-coded labels were then compared to the GPT-generated labels. Of the 300 double-coded reviews, 255 (85%) received identical theme assignments.

Finally, the 2622 Fitbit review comments categorized into the 14 affordances were reviewed by 1 author. This resulted in 1182 comments being retained for relevance.

### Participants and Recruitment Procedures

Before launching the main study, we carried out a pilot survey between June 1, 2022, and July 5, 2022. The participants were recruited through a university Sona Systems participant pool and via social media platforms (Facebook [Meta Platforms Inc] and Reddit [Reddit Inc]). In total, 403 individuals responded. After incomplete submissions, responses that failed validation checks, and cases showing inattentive patterns (such as straight-lining or unusually short completion times) were excluded, 81 usable responses remained. These participants were aged 18 to 52 years, with 318 (78.9%) participants being aged ≥25 years. Insights from this pilot, including feedback on the chosen affordances and the comments written in the “other” category, provided evidence supporting the adequacy of the survey instrument.

For the main study, we determined the required sample size via the formula by Gaskin [29]. Because social media recruitment produces a high rate of invalid responses, we opted to use Prolific (Prolific Academic Ltd), a crowdsourcing platform recognized for providing higher-quality data [30]. A prescreening survey was first distributed to 1300 participants, of whom 622 (47.8%) current adult US-based Fitbit users who engaged in exercise were identified. Of these 622 users, 506 (81.4%) completed the main survey. After applying quality control measures, including attention checks, of the 622 users, 442 (71.1%) valid responses were retained for analysis.

### Ethical Considerations

Both the pilot and main studies received approval from the institutional review board (IRB-22-0351) of the Worcester Polytechnic Institute and were conducted in accordance with ethical guidelines for human subject research. The participants provided informed consent electronically at the beginning of each survey, where they were informed about the study’s purpose, the voluntary nature of participation, and their right to withdraw at any time. The consent form also noted that the data would be stored securely and accessible only by the research team. All the responses were collected anonymously.

For the pilot study, student participants recruited through the university’s local participant pool received 0.5 course credit as an incentive, whereas participants recruited through social media received an opportunity to enter a raffle for 1 of 10 US $10 prizes as an incentive. The distribution of incentives was managed by one of the authors. For the main study, all recruitment and distribution of incentives were managed by Prolific. The participants recruited for the prescreening survey received a flat rate of US $0.18 as an incentive. Among these participants, those who were eligible to take part in the actual study (current Fitbit users who engaged in exercise) received a flat rate of US $6 as an additional incentive.

## Results

### Descriptive Statistics

The main study sample demographics are shown in Table 2.

All participants were current Fitbit users with varying use patterns, as shown in Table 3.

**Table 2:** Demographics of the main study sample based on an online survey of US-based Fitbit users who engaged in regular exercise (N=442).

		Participants, n (%)
**Gender**		
	Man	221 (50)
	Woman	212 (47.9)
	Transgender	4 (0.9)
	Nonbinary	5 (1.1)
**Age (y)**		
	18-24	31 (7)
	25-34	146 (33)
	35-44	137 (31)
	45-54	57 (12.9)
	55-64	48 (10.9)
	65-74	18 (0.1)
	≥75	5 (1.1)
**Race**		
	Asian	35 (7.9)
	Black	40 (9)
	White	358 (80.9)
	Other	9 (2.1)
**Employment status**		
	Employed full time	309 (69.9)
	Employed part time	62 (14)
	Not in paid work (eg, homemaker, retired, or disabled)	40 (9)
	Unemployed (and job seeking)	18 (4.1)
	Other	13 (2.9)

**Table 3:** Use patterns of the main study sample based on an online survey of US-based Fitbit users who engaged in regular exercise (N=442).

		Participants, n (%)
**Frequency of Fitbit app use**		
	Daily	194 (43.9)
	4-6 times a week	137 (31)
	2-3 times a week	93 (21)
	Once a week	9 (2)
	Less than once a week	9 (2)
**Duration of Fitbit app use**		
	<1 month	0
	Between 1 week and 1 month	0
	1-3 months	18 (4.1)
	4-6 months	40 (9)
	7-9 months	22 (5)
	10-12 months	35 (7.9)
	>12 months	327 (74)
**Duration of premium use**		
	Never	88 (19.9)
	Up to 3 months or free trial	110 (24.9)
	4-6 months	44 (10)
	7-12 months	58 (13.1)
	>12 months	142 (32.1)
**Device used**		
	Phone	177 (40)
	Fitbit wearable device	181 (41)
	Smartwatch (such as Samsung Galaxy Watch and Apple Watch)	49 (11.1)
	Laptop or desktop computer	35 (7.9)
**Exercise engaged in**		
	Running	106 (24)
	Walking	274 (62)
	Biking	22 (5)
	Swimming	5 (1.1)
	Spinning	4 (9)
	Hiking	13 (2.9)
	Yoga	5 (1.1)
	Other	13 (2.9)

### Common Fitbit Actualized Affordances

Common affordances discovered in the fitness app literature, as presented with definitions in Table 4, were actualized by the participants in this study via Fitbit. However, as per the arguments in this study, users’ actualizations may vary from one fitness app to another. When Fitbit was used, as shown in Table 5, the most frequently actualized affordances among users were updating (n=351, 79.4%; 351 participants and 749 review mentions; total=1100) and reminding (n=319, 72.2%; 319 participants and 143 review mentions; total=462). These findings indicate that users rely primarily on Fitbit to maintain awareness of their activity status and receive prompts that help sustain exercise routines. Other affordances also demonstrated meaningful engagement: competing (99, 22.4%; 99 participants and 88 review mentions; total=187) and rewards (n=133, 30.1%; 133 participants and 32 review mentions; total=165) highlight the motivational role of competition and incentives, whereas comparing (n=151, 34.16%; 151 participants and 8 review mentions; total=159) and guidance (n=118, 26.7%; 118 participants and 25 review mentions; total=143) emphasize the app’s instructional and benchmarking functions.

Additional affordances were less prevalent but still notable: searching (n=135, 30.5%; 135 participants and 2 review mentions; total=137), encouraging (n=75, 16.9%; 75 participants and 19 review mentions; total=94), and watching others (n=68, 15.4%; 68 participants and 3 review mentions; total=71). Finally, recognizing (n=58, 13.1%; 58 participants and 1 review mention; total=59) and self-presentation (n=47, 10.6%; 47 participants and 4 review mentions; total=51) were the least actualized, reflecting more specialized uses such as acknowledgment and image projection. Overall, the results show that Fitbit affordances extend beyond core tracking functions to include social, motivational, and comparative features, although these were less consistently used across the user base.

**Table 4:** Definitions of common affordances based on the fitness app literature.

Common affordance	Definition
Comparing	Allows users to evaluate their performance relative to that of others [11,17,31]
Guidance	Offers structured instructions or exercise recommendations that help users perform activities effectively [17,31]
Self-presentation	Allows users to publicly share achievements or statuses, facilitating impression management and self-image [17]
Rewards	Provides recognition in the form of badges, points, or virtual incentives, reinforcing desired behaviors [11,17]
Recognizing	Facilitates acknowledgment or praise from others for achievements, enhancing validation and social reinforcement [11]
Encouraging	Permits users to receive motivational messages from others, facilitating social support [11]
Competing	Enables users to engage in challenges or contests, fostering motivation through social rivalry [11]
Watching others	Allows users to observe others’ activities or progress [17]
Reminding	Provides timely prompts or reminders for workouts and goals, supporting consistency and habit formation [11]
Updating	Enables continuous self-tracking and progress awareness [11]
Searching	Provides tools to look up workouts, information, or content, enabling discovery and information retrieval [11]

**Table 5:** Common affordance actualizations uncovered through the online survey and supported by Fitbit app reviews.

Affordance	Actualization via survey (N=442), n (%)	Actualization via Fitbit reviews—relevant comments (n=2648 Fitbit review comments in total), n/N (%)	Total actualizations (survey+reviews)
Updating	351 (79.4)	749/1124 (66.6)	1100
Reminding	319 (72.2)	143/245 (58.4)	462
Competing	99 (22.4)	88/129 (68.2)	187
Rewards	133 (30.1)	32/55 (58.2)	165
Comparing	151 (34.2)	8/27 (29.6)	159
Guidance	118 (26.7)	25/143 (17.5)	143
Searching	135 (30.5)	2/18 (11.1)	137
Encouraging	75 (17)	19/475 (4)	94
Watching others	68 (15.4)	3/3 (100)	71
Recognizing	58 (13.1)	0/1 (0)	58
Self-presentation	47 (10.6)	1/25 (4)	51

### Novel Actualized Affordances

The participants also selected an option in the survey to write down different affordances that they had actualized via Fitbit. This resulted in 158 written responses (affordances). Some of the affordances written were similar to those listed in the survey but were written by participants with more use specifications. For example, while one of the choices for using Fitbit in the survey was “to update me with the status of my exercise,” some participants selected “others” to write “to see my progress for the day.” We excluded responses related to common affordances (n=129, 81.6%) and were left with 29 responses for further analysis.

We analyzed these 29 responses based on the 3 affordance principles we followed [23]. This resulted in the identification of 3 novel affordances, as defined in Table 6 and shown with user comments and quotes from the survey in Table 7. For example, as a purpose of using Fitbit, some participants wrote “to hold myself accountable.” This finding indicated that, using Fitbit, users built a sense of accountability toward exercising. In other words, Fitbit affords users the feeling of being accountable as a motivation to accomplish their regular exercise. These 3 affordances were used as themes to be uncovered within users’ comments on Fitbit.

As shown in Table 8, novel affordances included encouraging others (n=14, 3.2%; 14 participants and 1 review mention), accountability (n=3, 0.7%; 3 participants and 9 review mentions), and self-comparison (n=3, 0.7%; 3 participants and 5 review mentions).

**Table 6:** Definitions of novel affordances.

Affordance	Definition
Accountability	A sense of accountability for pursuing their exercise using various features such as today’s activity, cheer, and friends
Self-comparing	Assessing oneself against specific criteria or standards to gain insights into one’s strengths, weaknesses, areas for improvement, and overall effectiveness
Encouraging others (the opposite of receiving encouragement)	Offering support, motivation, or positive feedback to someone to boost their confidence, inspire action, or reinforce desirable behavior

**Table 7:** Participant and user quotes reflecting the novel affordances.

Affordance	Actualization via survey (N=442)	Actualization via Fitbit reviews (n=2648 Fitbit review comments)
Accountability	“To hold myself accountable” “To hold each other accountable” “To hold myself accountable to good exercise”	“Very easy way to check my progress and hold myself accountable to my fitness goals.” “Let us me know to move, check my blood sugar & monitors my progress. Makes me accountable!”
Self-comparing	“To compare myself with my past performance” “I like to see my overall trend for the week” “Love to compare what I do week to week”	“My favorite app! I have 10 years of step data and can look back to see how various activities have increased my steps over the years. All the data is still there through several Fitbit trackers and 3 phones.” “Do not realize how much walking I’m doing. However, it great to see I’m no longer a couch potato.”
Encouraging others	“To encourage my friends when competing” “To encourage others’ exercise” “To give other people motivation”	“Love the Fitbit app and all it has to offer helps me stay on track and encourage others to challenges.”

**Table 8:** Novel affordance actualizations uncovered through the online survey and supported via Fitbit app reviews.

Affordance	Actualization via survey (N=442), n (%)	Actualization via Fitbit reviews—relevant comments (n=2648 Fitbit review comments), n/N (%)	Total actualizations (survey+reviews)
Accountability	3 (0.7)	9/112 (8)	12
Self-comparing	3 (0.7)	5/81 (6.2)	8
Encouraging others	14 (3.2)	1/1 (100)	15

## Discussion

### Principal Findings

Although prior studies have identified several frequently actualized affordances in fitness apps, much of this work examines multiple platforms collectively and relies largely on affordances already established in the literature. This broad approach obscures meaningful app-specific differences and constrains the discovery of novel affordances that may emerge from users’ diverse goals and interaction patterns. As a result, existing research provides an incomplete account of how fitness apps generate value in real use contexts.

This study explored how Fitbit users actualized both common and novel affordances for the sake of deeper understanding of fitness app true value. The study integrated insights from survey responses and app store reviews and used established guidelines to uncover the affordances. The findings reaffirm that certain affordances, particularly updating and reminding, are central to users’ engagement. These affordances align closely with prior research emphasizing self-monitoring and feedback mechanisms as key drivers of continued use in fitness app contexts [32-35]. By enabling users to track their current activity and receive prompts to sustain routines, Fitbit fulfills core behavioral support functions that encourage habit formation and long-term adherence [10,11,15-17].

While updating and reminding dominated the value that users derived from Fitbit, other affordances played meaningful roles in shaping some of the users’ engagement. Competing and rewards, for instance, highlighted the motivational force of gamification, consistent with previous work demonstrating the appeal of competitive challenges and incentive-based reinforcement in fitness technologies [36]. Similarly, comparisons and guidance emphasize the importance of reference points and instructional support, showing that users draw value not only from tracking but also from situating their performance against that of others and learning how to optimize their efforts [37]. These findings resonate with affordance actualization theory, which stresses that technologies are appropriated in ways that reflect both design intent and users’ personal goals.

Although less frequent, affordances such as encouraging, watching others, recognizing, and self-presentation revealed meaningful but more specialized social and motivational dimensions of use. Encouragement and recognition, in particular, pointed to the social support embedded in fitness apps even if these affordances are not commonly actualized. Self-presentation emerged as the least actualized affordance, suggesting that most Fitbit users may not use the app primarily for impression management. These findings indicate that not all the possible affordances will be actualized by all fitness apps users. As this study showed, some affordances were barely actualized by users. These findings may contrast with those of prior work that documents high user reliance on socially oriented fitness app affordances, including competition and peer encouragement [10,18], suggesting that affordance actualization mechanisms are not uniform across fitness technologies. This could be due to their type of users or the way in which these fitness apps are designed. Consequently, the study findings suggest that affordance actualization should be examined at the level of the individual technology as even similar technologies (eg, fitness apps) demonstrate distinct actualization patterns shaped by their design configurations and user populations.

Most fitness app studies have applied common affordances [15,16,18-20], but this study revealed several novel affordances contributing to the niche literature [10,11,17,31]. The *emergent* affordances were accountability, self-comparison, and encouraging others. These affordances represent additional ways in which a subset of users interact with Fitbit. For example, accountability reflects users’ desire to justify actions to themselves, aligning with broader theories of self-regulation [25]. Previous studies have discovered that comparing oneself to others is an affordance of fitness apps [11,17]. Self-comparison extends the comparison literature by shifting the frame of reference from others to one’s past self, highlighting reflective, longitudinal self-evaluation [38]. These affordances illustrate the nuanced ways in which users may reinterpret features to satisfy individualized goals.

It is important to interpret the novel affordances with caution. These emergent themes were reported by a small subset of participants and, therefore, should not be considered dominant affordances of the Fitbit app. Rather, they represent preliminary evidence of user-technology relations that extend beyond commonly applied, predefined affordance taxonomies. Although low in frequency, such responses are theoretically meaningful, as affordance actualization theory acknowledges that users may perceive and enact unintended or highly individualized action possibilities depending on personal goals and situational contexts [13,21]. These emergent patterns, while not widespread, highlight opportunities for future research to validate and elaborate on these affordances using larger samples and person-centered analytical approaches.

From a design perspective, the identification of these novel affordances suggests actionable avenues for developers to support users who seek stronger behavioral reinforcement and self-referential goal alignment. For example, to better enable the accountability affordance, fitness platforms could implement a commitment contract module (eg, structured pledges such as “I commit to...” with self-defined consequences) and provide an accountability dashboard that transparently tracks completed vs missed exercise sessions. To facilitate the self-comparison affordance, apps may incorporate temporal self-evaluation features such as “Past me vs now” comparison cards, digital snapshot summaries, and personal improvement challenges that benchmark current performance against a user’s historical averages (eg, past–7-, 30-, or 365-day activity baselines). Finally, to support the affordance of encouraging others, platforms can introduce encouragement-based interactive elements, including peer cheer buttons, shareable motivation templates, and encouragement badges or kudos that users can award to friends or groups to reinforce social support behaviors.

Taken together, these design examples demonstrate how specific features can be engineered to strengthen the perception and actualization of emerging affordances, particularly for users who rely on digital tools not just for tracking activity but also for sustaining routines, reflecting on personal progress, and motivating others. This reinforces the importance of examining affordances within the context of a single technology ecosystem and tailoring future affordance research to behavioral patterns, motivational drivers, and downstream outcomes that reflect real user goals rather than only surface-level feature descriptions.

### Limitations and Future Research

The first limitation of this study relates to the sample composition and self-report nature of the data. Although the dataset included a robust number of respondents (N=442), the sample consists exclusively of current US-based Fitbit users, limiting generalizability to broader global populations or non-Fitbit fitness app users. Most participants were White (358/442, 80.9%), employed (371/442, 83.9%), and aged 25 to 44 years (283/442, 64%), which may underrepresent the perspectives of older adults, unemployed individuals, or ethnically diverse groups, segments that may perceive or actualize affordances differently. Additionally, while 74% (327/442) reported more than 12 months of app use and 43.9% (194/442) used Fitbit daily, exercise behavior regularity was not directly measured beyond self-identification, meaning that the group-based causal inferences about exercise consistency or app-enabled intervention needs remain interpretive rather than behavioral. Finally, only 41% (181/442) used a Fitbit wearable device, and 62% (274/442) tracked walking activities, suggesting that affordance experiences may reflect tracking-light or single-activity use patterns, potentially overlooking affordances that emerge in more intensive, multiactivity, or device-dependent exercise contexts. Therefore, future studies should replicate the model with diverse, international, and behavior-verified samples and explore predictors and outcomes of affordance actualization to deepen explanatory insights.

The second limitation is that review comments were analyzed primarily to corroborate the survey findings rather than identify additional affordances. Future research could extend this approach by systematically examining other Fitbit affordances within user reviews, thereby offering a more comprehensive understanding of the range of affordances that a technology such as Fitbit offers to users.

The third limitation is that, although demographic and use characteristics were collected, several variables exhibited substantial distributional imbalance (eg, duration of use and exercise type), limiting the feasibility of fine-grained subgroup analyses. Future research with more balanced and behavior-verified samples is needed to examine the heterogeneity of Fitbit affordance actualizations in greater depth.

### Conclusions

This study shows that Fitbit users actualize a range of common and novel affordances, highlighting how individuals engage with fitness technology in diverse and app-specific ways. By identifying common actualized and emerging affordances, the findings reinforce the importance of examining affordance actualization within a single technological context. Overall, this study advances understanding of how fitness apps create value for users and offers direction for designing features that better support sustained engagement and healthier behavior.

## Appendix

Multimedia Appendix 1 Base prompt used for generative pretrained transformer–based thematic classification.

Multimedia Appendix 2 Sample review inputs and generative pretrained transformer outputs.

## Abbreviations

CHERRIES: Checklist for Reporting Results of Internet E-Surveys
GPT: generative pretrained transformer

## Data Availability

The datasets generated or analyzed during this study are not publicly available due to institutional review board but are available from the corresponding author on reasonable request.
