# Supplementary material for: Exploring Common and Novel Actualized Affordances of Fitbit: Mixed Methods Study
Source: JMIR Hum Factors. 2026 Feb 18;13:e85412. doi: 10.2196/85412 (PMC12961385; doi:10.2196/85412)
Supplement: Multimedia Appendix 1 [file humanfactors_v13i1e85412_app1.docx]

Appendix A. Base Prompt Used for GPT-Based Thematic Classification

Prompt utilized for Review Classification

Your task is to classify each user review into a high-level theme.

There are 14 predefined themes. If the review clearly fits one of them, use that theme exactly as written. If the review does not match any of the 14 themes, generate a new theme that is a single word, broad and general in scope, distinct from the existing themes, and created only if truly necessary.

Existing themes (affordances)

Comparing – Comparing performance with others

Guidance – Getting instructions or help on how to exercise

Self-presentation – Presenting oneself as active or fit

Rewards – Receiving points, badges, or achievements

Recognizing – Getting praise, likes, or recognition from others

Encouraging – Being motivated or encouraged by others

Competing – Competing with others or tracking ranks

Watching others – Observing or following others’ activities

Reminding – Getting reminders to exercise or move

Updating – Tracking progress or status

Searching – Looking for information or exercise routines

Accountability – Holding oneself or others responsible

Self-comparing – Comparing with past performance

Encouraging others – Motivating or cheering others

New theme rules (non-affordances)

If the review concerns content not covered by the themes above, generate a new theme name using a single word (e.g., “Sleep,” “Hydration,” “Nutrition”). The new theme should be general enough to cover multiple similar reviews and should not represent a narrow or highly specific concept.

Review:

“{text}”

Respond with only the theme name. Do not include explanations or additional text.
