# Supplementary material for: Exploring Common and Novel Actualized Affordances of Fitbit: Mixed Methods Study
Source: JMIR Hum Factors. 2026 Feb 18;13:e85412. doi: 10.2196/85412 (PMC12961385; doi:10.2196/85412)
Supplement: Multimedia Appendix 2 [file humanfactors_v13i1e85412_app2.docx]

Appendix B. Sample Review Inputs and GPT Outputs.

| Assigned Theme | Review Excerpt |
| --- | --- |
| Accountability | Keeps me accountable, at least to some degree. |
|  | Great way to hold myself accountable. |
|  | Amazing. Easy to use and has helped in so many ways holding me accountable for steps each day. Highly recommend. |
| Comparing | I like to keep track of my steps every day. It helps me to make sure I'm getting enough exercise, and my wife and I like to compare how each one of us is doing. |
|  | Used now for 5yrs, kept me active, I like to see how I compare to my dog and his tracker. Great to assess my sleep too. |
| Competing | I have never been so fit since buying my fit bit love the competitions Update!! I used to love the challenges. They kept me committed to being fit. Ever since they took them out I struggle to make 10k |
|  | I like my Fitbit app. Good to check on sleep, walk with a group of friends and try to beat their steps. |
| Encourage | I am 77. Fitbit tracked my food, water, and steps to help me go from 291 to 163 so far. It encouraged me with all the help and prompt from the worldwide fitbit community. I love it! |
|  | Fitbit with, the help of my Fit Friends and our Challenges, gave me the Motivation I needed to lose over 100 pounds while in my 60s. Without the Challenges, and with no access to my Fit Friends, thanks to Google, I'm struggling to keep the weight off. |
| Encouraging others | Love the fitbit app and all it has to offer helps me stay on track and encourage others to challenges. |
| Guidance | Really helpful tips and information. Very accurate information. Find to use. |
|  | Great and insightful good guide to be more aware about positive and healthy lifestyle |
|  | Very good to help guide healthy lifestyle process. Just needs some coding and UX tweaks! maybe some better food databases as well. but overall highly recommended, it can be life-changing! |
| Recognizing | Non was found |
| Reminding | It's a good reminder to keep moving. |
|  | I love my fit bit! I especially like the reminders to get my steps in and the celebratory fireworks that goes off when I reach them. It has helped me track my sleep as I am trying to improve the quality of sleep daily. |
| Rewards | I LOVE Fitbit! I love keeping up with my health, and Fitbit allows me to do it. I receive encouragement each day to reach my goals and am awarded badges when I do well. And the sleep part of the app is wonderful showing me my sleep habits and what I need to do for improvement. I will never be without my Fitbit. Everyone should have the watch and the app! |
|  | I really love my Fitbit versa 3 . I'm disabled due to cancer, and this device works well with Humana, so they know my activity that im getting in every day, and the awesome part is I get rewarded! Fitbit rocks!! |
| Searching | Lots of great workouts. Easy to search for the type and length of workout you want. |
|  | Easy to negotiate and get information I need to keep goals and continue to live my life better. |
| Self-comparing | I love tracking how hard I worked out. If my numbers are low I try to get better numbers the next day. |
|  | My favorite app! I have 10 years of step data and can look back to see how various activities have increased my steps over the years. All the data is still there through several Fitbit trackers and 3 phones |
|  | Do not realize how much walking I'm doing. However, it great to see I'm no longer a couch potato. |
| Self-presentation | I would like to share my activities. |
| Updating | Love it. I don't use all of the functions most of the time but I do track my steps daily and that's the most important thing to me. Gotta keep moving at 72 years of age!! |
|  | This has been an really good app for me to use as it keeps me updated with my health |
| Watching others | Just great to see how you're doing. |
|  | Easy to use. Good to see how your friends are stepping.  Really enjoy this app gives you everything you need and easy to use. Mostly I like how you can add friends and family to see how they all |
|  | do. UPDATE still enjoy my Fitbit put a lot of kilometers on Fitbit I have also upgraded my Fitbit twice. Third update may be in the future |
